# Anti-mullerian hormone levels before and after ovarian drilling in polycystic ovary syndrome: has this an effect on fertility?

**DOI:** 10.1186/s12958-022-01002-4

**Published:** 2022-08-30

**Authors:** Mojgan Javedani Masroor, Hossein Sheybani, Shiva Sheybani, Nastaran Abolghasem

**Affiliations:** 1 Shahid Akbar-Abadi Clinical Research and Development Unit, School of Medicine, Iran University of Medical Sciences, Tehran, Iran; 2 Clinical Research Development Unit, Imam Hossein Hospital, Shahroud University of Medical Sciences, Shahroud, Iran; 3 School of Medicine, Tehran University of Medical Sciences, Tehran, Iran; 4 Firooz-Abadi Hospital, Department of Gynecology, Iran University of Medical Sciences, Tehran, Iran

**Keywords:** Polycystic ovary syndrome, Anti-mullerian hormone, Laparoscopic ovarian drilling

## Abstract

**Background:**

Polycystic ovary syndrome (PCOS) is the most common endocrine, metabolic, and multi-causal disorder in the reproductive period with a possible genetic origin. Women with PCOS are characterized by oligo-ovulation, clinical or biochemical hyperandrogenism, and polycystic ovaries. Women with PCOS have an increased number of antral follicles. Anti-Mullerian hormone (AMH), a dimeric glycoprotein produced from the granulosa cells of the pre-antral and antral follicles, is elevated in PCOS. AMH has been implicated in two stages of follicle dysfunction that lead to the development of PCOS. The level of AMH decreases following ovarian drilling in patients with PCOS. The present study compared the level of AMH before and after Laparoscopic ovarian drilling (LOD) in patients with PCOS and its effect on fertility.

**Materials and methods:**

This cohort study was carried out on 84 women with PCOS who underwent LOD in Akbarabadi Hospital in Tehran in 2020. Demographic characteristics, AMH, and estradiol levels were determined before surgery and compared with the amount one week after surgery. The effect of AMH level on pregnancy rate was also evaluated.

**Results:**

The mean age of the patients was 29.01 ± 4.01 years. The mean Body Mass Index (BMI) of the patients was 26.33 ± 4.14 kg/m^2^. The results showed that the mean AMH level decreased significantly after ovarian drilling (*P*-value < 0.001). Menstrual cycle distribution was significantly different before and after LOD (*P* < 0.001). None of the variables had an effect on the pregnancy (*P*-value > 0.05). Oligomenorrhea in the previous menstrual period might cause AMH levels to increase by 3.826 units after LOD (*P*-value < 0.001).

**Conclusion:**

Measuring serum AMH concentration before treatment can be a useful tool to predict LOD outcomes. This can help in selecting the patient for treatment.

**Trial registration:**

The project was found to be under the ethical principles and the national norms and standards for conducting research in Iran with the approval ID and issue date of IR.IUMS.FMD.REC.1397.206 and 2018.08.26 respectively, which has been registered with the research project number 2766 in the Vice-Chancellor for Research and Technology Development of Iran University of Medical Sciences, School of Medicine, Tehran, Iran. URL: https://ethics.research.ac.ir/EthicsProposalView.php?id=34791.

## Introduction

Polycystic Ovarian Syndrome (PCOS) with a prevalence of about 4–18% is associated with menstrual problems, hyperandrogenism, and polycystic ovaries [1]. PCOS causes approximately 75% of infertility due to lack of ovulation [2]. According to a national study, the prevalence of PCOS is 14.2% in Iran [3]. In addition to infertility, this syndrome is associated with insulin resistance, hyperinsulinemia, hyperandrogenism, features of the metabolic syndrome, and an increased risk of diabetes [4].

Although the pathogenesis of this disease is complex and not fully understood, studies have shown that androgens and insulin are the main causes of this disease [5]. Insulin has major effects on the ovaries and the follicles. Hyperinsulinemia is associated with the under-developing growth of immature ovarian follicles [6]. Increased androgens and an inherent increase in the number of follicles in women with PCOS increase the production of Anti-Mullerian Hormone (AMH) [7].

People with PCOS have higher levels of testosterone, insulin, triglycerides, cholesterol, and lutein than healthy people. They also have lower levels of sex hormone-binding globulin (SHBG) and follicular growth hormone (FSH) than healthy people [7, 8]. Dyslipidemias are also a common finding in PCOS. Other findings in women with PCOS include an increase in the prevalence of hypertension, a higher incidence of atherosclerosis and cardiovascular disease, and an increased risk of myocardial infarction (about 7 times higher than the healthy people) [5]. The clinical association between hyperinsulinemia and anovulation associated with hyperandrogenism is well known worldwide and among all racial groups [5]. Due to the physical and mental problems following PCOS in women, this disease significantly reduces the quality of life [7]. These patients are at risk for various psychological disorders due to metabolic disorders and especially disorders in the level of sex hormones, especially testosterone [8–10].

The secretion of AMH decreases gradually during the stages following the growth of the follicle and is particularly impossible in follicles larger than 8 mm [8]. Serum AMH concentration is associated with the number of small follicles, followed by an ovarian reserve [9]. Animal studies show that AMH has an inhibitory effect on the uptake of primordial follicles, thereby preventing their rapid termination. It has also been shown that AMH reduces the sensitivity of follicles to circulating FSH and can play an important role in normal folliculogenesis [10, 11]. During follicular growth, when a follicle reaches a certain size (8 mm), AMH expression decreases, resulting in an increase in the sensitivity of the follicle to circulating FSH, thus, a decrease in AMH levels provides an opportunity for follicles to grow until ovulation. Previous studies have shown that women with polycystic ovary syndrome have a 2 to 3 times increase in their serum AMH, followed by a 2 to 3 times increase in the number of small follicles (2–5 mm) [12, 13]. Increasing the concentration of AMH affects the pathogenesis of polycystic ovary syndrome [14]. Studies show that AMH inhibits the aromatase enzyme, which reduces the production of follicular estradiol, and decreased estradiol levels may be associated with defects in dominant follicle selection [15].

Numerous studies have shown that nutritional status and obesity can affect AMH synthesis [13–15]. Some researchers have reported lower levels of AMH in obese women and found an inverse relationship between AMH and body mass index (BMI) [17, 18], while others did not report a relationship between nutritional factors, BMI, and AMH [14, 15]. Due to the high level of AMH in the patients with PCOS and the possibility of developing ovarian hyperstimulation during infertility treatment in these patients, we decided to compare the level of AMH before and after Laparoscopic Ovarian Drilling (LOD) in these patients and its effect on fertility.

## Materials and methods

The present study was a cohort study consisting of 84 women with PCOS who underwent laparoscopic ovarian drilling in Akbarabadi hospital in Tehran in 2020. The sample size was calculated according to the La Marca et al. study [15] and based on the following formula, considering alpha as 0.05, d as 0.05, and P as 0.3:

$$\frac{{(\mathrm{Z}1-\mathrm{\alpha }/2)}^{2} [(\mathrm{P }(1-\mathrm{P})]}{{(\mathrm{d})}^{2}}$$

Inclusion criteria were the PCOS patients based on the Rotterdam criteria (2003) [16] and candidates for laparoscopic ovarian drilling treatment. Exclusion criteria were the patients who were not willing to participate in the study and follow-up visits. AMH and estradiol levels of the patients were calculated before the surgery and one week after the surgery. Also, the success rate of fertility was assessed within the twelve months after the surgery, which was indicated by Intrauterine insemination (IUI) or in vitro fertilization (IVF).

### Ethical considerations

The research followed the Tenets of the Declaration of Helsinki. This study was approved by the ethics committee of the Iran University of Medical Sciences (IR.IUMS.FMD.REC.1397.206). Accordingly, informed consent was obtained from all the patients.

### Statistical analysis

Descriptive results were presented as mean ± standard deviation (SD) or percentage. An independent t-test was used to compare the two means and in case of abnormal data distribution, the Mann–Whitney U test was used. Also, McNemar's test was used to investigate the differences between binary qualitative variables. One-way analysis of variance was used to compare more than two means. Logistic regression and linear regression models were used to control the confounders. A *P*-value less than 0.05 was considered statistically significant. All data were analyzed using SPSS software version 21.

## Results

The mean age of the patients was 29.10 ± 4.01 years. The mean BMI of the patients was 26.33 ± 4.14 kg/m^2^. Most (41%) of the women had normal BMI. The comparison of mean AMH before and after ovarian drilling was performed using paired t-test which showed that the mean AMH after LOD had a significant decrease (*P* < 0.001) (Table 1).

**Table 1:** Mean and standard deviation of AMH before and after ovarian drilling

Variable	Mean	Number	SD	Mean deference	*P*-value
AMH before LOD	8.8188	78	6.16	3.02	< 0.001
AMH after LOD	5.7936	78	3.65		

A comparison of menstrual cycle distribution before and after ovarian drilling was performed using McNemar's test which showed that there was a significant difference between menstrual cycle distribution before and after ovarian drilling (*P* < 0.001) (Table 2).

**Table 2:** Menstrual cycle distribution before and after ovarian drilling

Menstrual cycle		After		Total
		Regular pattern	Oligomenorrhea	
Before	Regular pattern	44	0	< 0.001
	Oligomenorrhea	18	16	
Total		62	16	

The relationship between positive or negative pregnancy with age, BMI, AMH, and the menstrual cycle was performed using logistic regression analysis, which showed that none of the variables had an effect on pregnancy (*P*> 0.05 (Table 3).

**Table 3:** Logistic regression analysis of the relationship between positive or negative pregnancy dimensions with age, BMI, AMH dimension, and menstrual cycle

Variables	B	Std. Error (SD)	Odds Ratio (OR)	95% Confidence interval		*P*-Value
				Lower	Upper	
Age	-0.064	0.079	0.938	0.804	1.095	0.418
BMI	0.048	0.071	1.050	0.913	1.206	0.495
AMH after	-0.058	0.087	0.944	0.796	1.120	0.507
Menes after	1.916	1.178	6.794	0.676	68.311	0.104

The relationship between mean AMH after drilling with BMI category was investigated using a one-way analysis of variance. The results showed that the mean AMH after ovarian drilling was not significantly different in the BMI categories (*P* = 0.181) (Table 4).

**Table 4:** Mean AMH after ovarian drilling across BMI category

BMI (kg/m^2^)	Number	Mean	SD	95% Confidence Interval for Mean		*P*-value
				Lower Bound	Upper Bound	
18.5–25	32	5.13	3.37	3.9156	6.3469	0.181
25–30	31	6.73	4.00	5.2651	8.2058	
30–35	15	5.26	3.25	3.4567	7.0633	
Total	78	5.79	3.65	4.9695	6.6177	

The relationship between AMH after ovarian drilling with the variables of age, BMI, and the previous menstrual cycle was examined using linear regression. Age and BMI variables were not significantly associated with the previous menstrual cycle (*P* > 0.05) (Table 5).

**Table 5:** Linear regression results to evaluate the relationship between AMH after ovarian drilling with age, BMI, and previous menstrual cycle

Variables	Unstandardized Coefficients		95.0% Confidence Interval		*P*-value
	B	Std. Error	Lower Bound	Upper Bound	
Constant	3.989	3.271	-2.529	10.506	0.227
Age (year)	-0.100	0.091	-0.283	0.082	0.276
BMI (kg/m^2^)	-0.029	0.093	-0.214	0.156	0.754
Previous menstrual cycle	3.826	0.761	2.310	5.343	< 0.001

## Discussion

In the present study, we compared the level of AMH before and after laparoscopic ovarian drilling in patients with PCOS and its effect on fertility**.**

In a prospective cohort study performed by Elmashad et al., a significant decrease in serum AMH levels of the patients with PCOS was observed in the post-treatment phase and the final result indicates the role of AMH before laparoscopic ovarian drilling in predicting outcomes [17]. The results of this study were consistent with our study.

In a prospective cohort study performed by Farzadi et al. in Iran on 30 women with PCOS who underwent laparoscopic ovarian drilling, it was reported that there was a significant decrease in the serum AMH level in the post-treatment phase. The mean AMH before treatment was 8.4 ng/ml, one week, three months, and six months later were 7.5 ng/ml, 7 ng/ml, and 7.7 ng/ml respectively, indicating that this treatment did not cause a change in ovarian reserve [18].

A review study by Amer et al. in the UK on AMH levels in women with PCOS under laparoscopic ovarian drilling found a significant reduction in serum AMH levels by 2.13 ng/ml in these patients. In the post-treatment phase, it is observed that it is not clear whether this reduction is only within normal limits, such as in women without PCOS, or reduces ovarian reserve [19]. The results of this study were consistent with the present study.

A study done by Abu Hashim et al. in Egypt on serum AMH levels in women with PCOS under laparoscopic ovarian drilling in unilateral and bilateral cases reported a decrease in serum AMH levels in unilateral and bilateral laparoscopic cases [20]. This study also had consistent results with the present study.

An analytical study was conducted by Giampaolino et al. in Italy on 123 women with PCOS under laparoscopic ovarian drilling and 123 women with PCOS under Transvaginal hydrolaparoscopy (THL). Both methods showed that the rate of reduction was similar in the two methods [21].

In a prospective cohort study conducted by Paramu et al. in India on 30 women with PCOS under laparoscopic ovarian drilling, there was a significant reduction of up to 33% in the serum AMH level in the post-treatment phase [22]. The results of Paramu et al. and Gaafar et al.’s studies were consistent with the present study [22, 23].

Previous studies have concluded that a decrease in AMH after LOD should be considered a normalization process rather than a pathological decrease in ovarian reserve. Mild ovarian injury inefficiencies in performing LOD with four to five holes in the ovary are more effective than performing only two or fewer holes [22, 24, 25].

It is suggested that the increase in AMH levels in patients with PCOS is due to an increase in the number of primary antral follicles [26]. However, other reports have shown that the increase in AMH concentration is mainly due to the increase in AMH production by each follicle and is not just the result of an increase in the number of follicles [27]. AMH may be a marker of ovarian aging because it is associated with the number of primary antral follicles which indicates the size of the follicle rest chamber, so if AMH levels decrease, the ovarian reserve may be compromised [28].

The question is whether laparoscopy affects ovarian tissue and reduces ovarian reserve. Weerakiet et al. examined changes in serum AMH levels before and after LOD [29]. They found that the mean changes in serum levels of AMH in 21 patients with PCOS were three days after LOD, which was not statistically significant. In our study, mean levels of AMH did not change significantly before laparoscopy, 1 week, 3 months, and 6 months after laparoscopy.

AMH is considered an important marker of ovarian reserve [30]. Serum AMH is 2–4 times higher in women with PCOS than in healthy women. This is because PCOM ovaries show the number of small antral follicles producing AMH [31] and increase production in granulosa cells [32]. An acceptable explanation for the decrease in serum AMH after LOD could be the effect of heat damage, which reduces its production from the granulosa cells of the primary, pre-abdominal, and small antral follicles [20].

## Conclusion

Based on the results of this study, it seems that measuring serum AMH concentration before treatment can be a useful tool in predicting LOD outcomes. This also can help in choosing a suitable patient for the treatment. Further studies are needed to determine more accurately whether AMH is the cause or outcome of ovulation after surgery.

## Data Availability

All data generated or analyzed during this study are included in the manuscript.

## Abbreviations

AMH: Anti-Mullerian hormone
BMI: Body Mass Index
FSH: Follicular growth hormone
IUI: Intrauterine insemination
IVF: In vitro fertilization
LOD: Laparoscopic ovarian drilling
OR: Odds ratio
PCOS: Polycystic ovary syndrome
SHBG: Sex hormone-binding globulin
SD: Standard deviation
THL: Transvaginal hydrolaparoscopy
